# Supplementary material for: Community perceptions and acceptance of ivermectin for malaria control on Sumba Island, Indonesia
Source: PLoS One. 2026 Feb 13;21(2):e0326646. doi: 10.1371/journal.pone.0326646 (PMC12904417; doi:10.1371/journal.pone.0326646)
Supplement: S2 File — Qualitative research tools. https://doi.org/10.5281/zenodo.17769585. (DOCX) [file pone.0326646.s002.docx]

**Sumba Livestock Ivermectin for Malaria Control (SLIM)**

**STUDY CODE**

**FOCUS GROUP DISCUSSION GUIDE**

**After signing consent form, start recording**

| **General information** | |
| --- | --- |
| Audiences group: | Gender:  # of male: __  # of female: ___ |
| Date : [__\|__]/[__\|__]/[__\|__]  DD MM YY | |
| Location: | |
| **Introduction** | |
| Goal: We are interested to learn more about your experience about malaria, and thoughts about how it can be prevented in your community. In particular we are interested in your thoughts about the ‘SLIM study’ to prevent malaria by giving your animals medicine which kills the mosquitoes. | |
| **Questions:** Use of this guide is expected to be flexible for Focus group discussion. The guide is expected to help group discussion stay within the confines of the themes guided by the research question. Questions included below are only examples and should be adapted during the group discussion. Probes and potential questions under the themes can be re-phrased and asked in ways appropriate to the main concept. The researcher may add questions/probes to collect additional and important information | |
| **Target audiences**   1. 2 focus groups: 2. Each group will have 8-10 members   **Criteria**  For selecting target audiences in focus group discussion, we will select community members who are:   1. ≥ 18 years old 2. Have livestock animals in their wider family 3. Give consent to participate 4. Live in the study district   **Note for facilitators**   - Clear introduction: Purpose of FGDs, name of facilitator and any SLIM team members present, name of participants - Ask them to respect each other’s opinions - CONFIDENTIALITY - The FGD will be recorded for research use only. Aggregated and de-identified data will be shared with study team and for publication. - Consent – a participant may choose not to answer but their information can’t be withdrawn from the data once it’s spoken. Reassure that no names will be linked to the data. - Create a comfortable atmosphere for all participants - Remember not to use leading questions, select power question rather than weak questions for FGDs   **Questionnaires**  **PART 1: OPEN ENDED NARRATIVE OF MALARIA EXPERIENCES**  Goal: to gain a narrative of experiences from the participant’s point of view prior to any focused questions to gain a picture of what their experiences include   1. To start, we would like to know more about your experiences of malaria, yourself and your family, and in your community. After, we will ask more focused questions but to begin, please tell us how malaria has affected your life. Please start the story where you like and take as much time as you need.     Notes for facilitator can apply life mapping tools    **Part 2: Impact of malaria on individual and community life**   1. How do you know if someone has malaria rather than another illness? 2. Which people in your community are most affected by malaria? 3. In what way are they affected? [Prompts - Income, unable to work, children off school] 4. What actions do you do when you or your family members are sick with malaria? 5. What are the main challenges for people when they/family member get sick with malaria? 6. Rank these health problems for your family / the whole community   Facilitator should use sorting cards. Have a multiple sets of cards – first get people to rank for their own family, then as a group for the wider community]. Cards include: malaria, skin diseases, TB, diarrhoea, fever, injuries, Covid-19, dengue, influenza, pregnancy related problems, childhood illnesses (measles etc), intestinal worms, snakebites, tetanus  **Part 3: Current preventative measures against malaria**   1. How do you think malaria is spread to people? [explore their knowledge of mosquito transmission]. 2. What is currently being done in your communities to reduce malaria? 3. Who is responsible for malaria control? Does it happen? Does it work? 4. What do individual families do to prevent malaria? 5. Has Covid-19 impacted malaria control measures?   **Part 4: Perceptions about SLIM study and potential of MDA ivermectin**  Notes for Facilitator: You may need to describe the SLIM study if participants aren’t familiar with it.   1. There is a medicine (Ivermectin) which can kill mosquitoes. It is a safe medicine for people and animals. How do you feel if we put this medicine into your cows/buffalo/goats to kill the mosquitoes that bite the animals? [Are they more sensitive about certain animals? How do they feel about injections?] 2. If we tell you that the medicine will also kill all the ticks and parasites in your animals does this make you less / more / no difference accepting of the medicine? 3. Who do you think should be asked to give this injection to your animals? 4. What do you think about injecting all the animals in the village to reduce the number of mosquitoes and reduce spread of malaria? 5. What do you think about orally treating all the people in your village with this medicine to kill mosquitoes and reduce spread of malaria? The medicine is safe for people and animals. [Explore whether they would be willing to be treated? Their children? Any people who shouldn’t have it?]   **Part 5. Community questions about the study** | |
| **Interview Ending** | |
| Thank you and stop recording | |
