# Supplementary material for: Community perceptions and acceptance of ivermectin for malaria control on Sumba Island, Indonesia
Source: PLoS One. 2026 Feb 13;21(2):e0326646. doi: 10.1371/journal.pone.0326646 (PMC12904417; doi:10.1371/journal.pone.0326646)
Supplement: S3 File — Qualitative research tools. https://doi.org/10.5281/zenodo.17769585. (DOCX) [file pone.0326646.s003.docx]

**Sumba Livestock Ivermectin for Malaria Control (SLIM)**

**STUDY CODE**

**Semi-structured interview – community**

In-person interviews will last between 60 minutes. Where face to face interviews are not possible due to Covid19 restrictions online/telephone interviews may be conducted over a series of shorter online interactions with the same participant. Questions might be re-phrased, when necessary, and additional topics and probes will be included, based on the responses of the interviewees, as well as information gathered during other data collection.

Use of this guide is expected to be flexible for semi-structured interviews. The guide is expected to help interviews stay within the confines of the themes guided by the research question. Questions included below are only examples and should be adapted during the interview. Probes and potential questions under the themes can be re-phrased and asked in ways appropriate to the main concept. The researcher may add questions/probes to collect additional and important information.

In general, all questions in this guide should be asked, however, the order can be edited to make the interview more conversational.

| **Ice breaker** |
| --- |
| **Informed consent**  If first interview, conduct full consent process; if subsequent interview, remind participant about nature of study and consent process. In all cases, ask participant if they have any questions about the study or their participation in this study. |
| **Participants’ information** |
| Participant ID [Given by study team]  Age  Gender  Occupation  Number of animals  Cattle :  Buffaloes :  Horses :  Village |
| **Interview part 1: Open ended narrative of malaria experiences** |
| [*Goal: to gain a narrative of experiences from the participant’s point of view prior to any focused questions to gain a picture of what their experiences include.]*   1. To start, we would like to know more about your experiences about malaria. After, we will ask more detailed questions but to begin, please tell us how malaria affects you, your family and your community. Please start the story where you like and take as much time as you need. 2. [*when they are finished*] Thank you for sharing your story. I would like to ask you more about [*insert 1-2 questions that you would like to probe on from their story*].   [*probe: during the narrative, try not to interrupt for details – note any questions you would like to probe on, use acknowledgement probes, e.g. ok, yes, mhhh and gentle probes, e.g. “is there anything else you would like to add” or “what else happened?” and then ask follow-up questions after.]* |
| **Interview part 2: How is malaria understood in different locales?** |
| Topics: malaria knowledge, health care seeking behaviour, information     1. Could you tell me more about what you know about malaria?    1. [*potential probes: where did it come from? How does it spread? Is it dangerous? Who gets it? Is anyone at more/less risk to acquire it? Add additional probes from survey results]* 2. What are the main symptoms of malaria? 3. Have you or anyone in your immediate family had malaria? [don’t ask this if it’s come up in Part 1]. 4. What does your family do if someone gets malaria? [probe: health seeking behaviour, barriers to getting professional help, self-treatment, non-medical treatments] 5. Is your family typical of your community in this regard or do others have different behaviours about malaria? 6. Where do you and your family obtain information about malaria? [*probes, if necessary*: *TV, social media (Facebook, Zalo), radio, friends, family, healthcare professionals]* 7. Could you tell me more about the types of information provided from these sources? [*probe: What are some of the exact messages or information (‘facts’) that you remember from these sources?]* 8. From your point of view, what are the main impacts of malaria in your community? [*probe: finiancial, time off work, fields, death, sickness of children..]*   What are the most important health problems in your community? How does malaria compare with the? [*probe about relative importance of these:* *malaria, skin diseases, TB, diarrhoea, fever, injuries, Covid-19, dengue, influenza, pregnancy related problems, childhood illnesses (measles etc), intestinal worms, snakebites, tetanus, rabies. Probe WHY some are more important?]* |
| **Interview part 3: Disease transmission, adherence to public health guidelines, and ad hoc harm reduction** |
| We have a few questions about various measures you and your family have taken in response to Malaria. By family, for these questions, we mean those who are living in your home with you.   1. Who all is living in your home right now? [*probe: # of people, generations*] 2. What do you and your family do to reduce the chances of getting malaria? [*probe: for each method, ask their perception of the effectiveness and how easy or difficult it is for them and their family to do it and why*] 3. Is there any community action to reduce malaria? Describe it. |
| **Interview part 4: Perceptions about SLIM study and potential of MDA ivermectin** |
| Notes for Facilitator: You may need to describe the SLIM study again if participants aren’t familiar with it.   1. There is a medicine (Ivermectin) which can kill mosquitoes. It is a safe medicine for people and animals. How do you feel if we put this medicine into your cows/horses/goats to kill the mosquitoes that bite the animals? [Probe: Are they more sensitive about certain animals? How do they feel about injections?] 2. If we tell you that the medicine will also kill all the ticks and parasites on your animals does this make you less / more / no difference accepting of the medicine? 3. Who do you think should be asked to give this injection to your animals? 4. What do you think about injecting all the animals in the village to reduce the number of mosquitoes and reduce spread of malaria? 5. What do you think about injecting all the people in your village with this medicine to kill mosquitoes and reduce spread of malaria? The medicine is safe for people and animals. [Probe: *Explore whether they would be willing to be injected? Their children? Any people who shouldn’t have it?]* |
| **Interview part 5: Wrap up** |
| 1. Is there anything else you would like to add to our discussion related to your experiences of Malaria? 2. Is there anything else you would like to ask about the SLIM study?   *Thank the participants for their time and arrange their compensation. Explain that they can contact the study representative [name] if they have further questions about the study.* |
