# Supplementary material for: Community perceptions and acceptance of ivermectin for malaria control on Sumba Island, Indonesia
Source: PLoS One. 2026 Feb 13;21(2):e0326646. doi: 10.1371/journal.pone.0326646 (PMC12904417; doi:10.1371/journal.pone.0326646)
Supplement: Attachment — Submitted filename: Response to reviewers_SLIMCE_Jan26.docx [file pone.0326646.s006.docx]

12 January 2026

Pyae Linn Aung 
Academic Editor 
PLOS ONE

**PONE-D-25-10247: Community perceptions and acceptance of ivermectin for malaria control on Sumba Island, Indonesia**

Dear Dr Pyae Linn Aung,

Thank you for the thoughtful review of our manuscript: Community perceptions and acceptance of ivermectin for malaria control on Sumba Island, Indonesia. We have revised the manuscript accordingly and have responded to the Editor’s suggestions below. We believe that these changes have substantially improved the paper.

Best wishes,

Mary Chambers on behalf of authors

**Manuscript edits. Line numbers correspond to the Revised manuscript with track changes.**

1. In the abstract, the Methods section should specify the target population and indicate the timeline of data collection (e.g., before or after the trial). The total sample size (n = 75) should be clearly categorized by data collection method (e.g., FGDs, IDIs, etc.).

**Edited as suggested**.

2. In the Introduction, it would be useful to acknowledge the recent resurgence of malaria in Indonesia. Additionally, please clarify that different Anopheles species exhibit varied host-seeking behaviors, some are zoophilic, while others are anthropophilic, given that the intervention in this trial targeted only animals.

**We have acknowledged the resurgence in malaria in Indonesia (line 65), and note the differing feeding patterns of Anophelese species. However, please note that this manuscript is focusing on the social science aspect of the study and the entomology data is presented in our first paper:** Kobylinski, K.C., et al., *Impact of standard and long-lasting ivermectin formulations in cattle and buffalo on wild Anopheles survival on Sumba Island, Indonesia.* Scientific Reports, 2024. **14**(1): p. 29770.

3. The Methodology section requires particular attention. The map should include a north arrow for clarity. The sampling strategy is unclear: please explain how participants were selected for each data collection method to ensure representativeness across study areas. Line 141 mentions gender balance, yet the majority of participants were male; moreover, Lines 148–149 indicate that women were uncomfortable participating in FGDs, which further limited female representation. This suggests that the findings primarily reflect male perspectives. Please describe the tools used for each data collection method, and consider attaching them as supplementary materials. Explain how responses were recorded, given that only one researcher conducted the data collection with assistance from a translator. Clarify the process of language translation and ensure the NVivo software is properly cited.

**Edits made as suggested and the gender of participants sampled by different methods is made clearer.**

**Tools have been made available** <https://doi.org/10.5281/zenodo.17769585>

4. Line 202: Participants included government officials, health cadres, community leaders, and livestock owners. Given their differing social roles and backgrounds, please explain how you ensured that all voices were equally represented during discussions.

**Edited as suggested. Line 193**

5. Line 204: Please clarify what is meant by “their experience.” For example, does this refer to years of livestock farming or another indicator?

**Edited as suggested. Line 264**

6. Although multiple qualitative methods were employed, the manuscript contains very few participant quotes. Including more direct quotations would strengthen the findings and illustrate key themes. Given the diversity of participants’ socioeconomic and demographic backgrounds, it would also be useful to identify the speaker type (e.g., community livestock owner, government official) when quoting, as their perspectives likely differ.

**Edited as suggested and more quotes have been included.**

7. Lines 290–310 discuss ivermectin MDA for humans. Please clarify how you ensured that participants understood the purpose of the drug when applied to humans, particularly since they observed its use in animals. This could have caused confusion about whether a drug given to animals is safe or appropriate for human use.

**Edited as suggested. Line 378-386.**

8. Several sentences should be reviewed for technical accuracy and precision. For example, Lines 24–25: not all Anopheles species transmit malaria. Lines 68–69: the claim that having many Anopheles species makes vector control difficult should be reconsidered or supported with evidence.

**Edited as suggested and made clearer.**

9. All references need to be revised to conform to the journal’s formatting requirements, both in the reference list and in-text citations. For instance, see Lines 371 and 433.

**Revised as suggested.**
